# Ceruloplasmin and Lipofuscin Serum Concentrations Are Associated with Presence of Hypertrophic Cardiomyopathy

**DOI:** 10.3390/biomedicines12081767

**Published:** 2024-08-06

**Authors:** Wiktoria Smyła-Gruca, Wioletta Szczurek-Wasilewicz, Michał Skrzypek, Ewa Romuk, Andrzej Karmański, Michał Jurkiewicz, Mariusz Gąsior, Tadeusz Osadnik, Maciej Banach, Jacek J. Jóźwiak, Bożena Szyguła-Jurkiewicz

**Affiliations:** 1 Student’s Scientific Society, 3rd Department of Cardiology, Faculty of Medical Sciences in Zabrze, Medical University of Silesia, 40-055 Katowice, Poland; wiktoriasmyla@gmail.com (W.S.-G.); jurkiewicz.m@yahoo.com (M.J.); 2 2nd Department of Cardiology and Angiology, Silesian Center for Heart Diseases, 41-800 Zabrze, Poland; 3 Department of Biostatistics, Faculty of Public Health in Bytom, Medical University of Silesia in Katowice, 40-055 Katowice, Poland; mskrzypek@sum.edu.pl; 4 Department of Biochemistry, Faculty of Medical Sciences in Zabrze, Medical University of Silesia, 40-055 Katowice, Poland; eromuk@gmail.com; 5 Department of Descriptive and Topographic Anatomy, Faculty of Medical Sciences in Zabrze, Medical University of Silesia, 40-055 Katowice, Poland; akarmanski@sum.edu.pl; 6 3rd Department of Cardiology, Faculty of Medical Sciences in Zabrze, Medical University of Silesia, 40-055 Katowice, Poland; m.gasior@o2.pl (M.G.); bjurkiewicz@sum.edu.pl (B.S.-J.); 7 Department of Pharmacology, Faculty of Medical Sciences in Zabrze, Medical University of Silesia, 40-055 Katowice, Poland; tadeusz.osadnik@icloud.com; 8 Cardiology and Lipid Disorders Clinic, Independent Public Health Care Institution “REPTY” Upper Silesian Rehabilitation Centre, 42-600 Tarnowskie Góry, Poland; 9 Polish Mothers Memorial Hospital Research Institute, 90-419 Łódź, Poland; maciej.banach@umed.lodz.pl; 10 Department of Hypertension, Medical University of Lodz, 90-419 Łódź, Poland; 11 Cardiovascular Research Centre, University of Zielona Gora, 65-046 Zielona Gora, Poland; 12 Department of Family Medicine and Public Health, Faculty of Medicine, University of Opole, 45-040 Opole, Poland; jacek.jozwiak.1234@gmail.com

**Keywords:** markers, stress oxidative, hypertrophic cardiomyopathy

## Abstract

Oxidative stress reflects an imbalance between the systemic manifestation of reactive oxygen species and cells’ ability to neutralize them by antioxidant systems. The role of oxidative stress in hypertrophic cardiomyopathy (HCM) is not fully understood. The aim of the study was to examine selected parameters of oxidative stress in patients with HCM compared to the control group. We enrolled 85 consecutive HCM patients and 97 controls without HCM. The groups were matched for sex, the body mass index, and age. Oxidative stress markers included superoxide dismutase (SOD), ceruloplasmin (CER), and lipofuscin (LPS). The median age of the HCM patients was 53 (40–63) years, and 41.2% of them were male. HCM patients, compared to the control ones, had significantly increased levels of CER and LPS. The areas under the receiver operating characteristics curves (AUC) indicated a good discriminatory power of CER (AUC 0.924, sensitivity 84%, and specificity 88%), an acceptable discriminatory power of LPS (AUC 0.740, sensitivity 66%, and specificity 72%), and poor discriminatory power of SOD (AUC 0.556, sensitivity 34%, and specificity 94%) for HCM detection. CER with good predictive strength, as well as LPS with acceptable predictive power, allows for HCM detection. The utility of SOD for HCM detection is limited.

## 1. Introduction

Hypertrophic cardiomyopathy (HCM) is a cardiac genetic disease mainly caused by mutations in sarcomere proteins and is characterized by left ventricular hypertrophy, diastolic dysfunction, and myocardial disarray [1,2,3]. The onset and presentation of HCM are extremely varied, ranging from asymptomatic mutation carriers through benign forms of HCM and ending with patients with severe heart failure [1]. Despite increasing knowledge of mutations associated with HCM, the exact pathway from the genetic defect leading to cardiomyopathy is complex and not fully understood. Taking into account the diverse pathophysiology of HCM and different clinical pictures of this disease, it seems reasonable to search for simple and cheap markers that can help select those patients who should undergo more detailed tests for the diagnosis of HCM [1,2,3,4,5].

Over the years, several studies have drawn attention to the relationship between disturbed oxidative–antioxidative balance and HCM [5,6,7,8]. Under physiological conditions, the reactive oxygen species (ROS) levels are strictly controlled by nonenzymatic and enzymatic antioxidant systems; nevertheless, under pathological conditions, excessive production of ROS exceeds the cell’s antioxidant capacity to their neutralization [9,10,11]. As a result, uncontrolled ROS production can cause cell dysfunction, changes in the structure, conformation and function of proteins, lipid peroxidation, and DNA mutagenesis and may lead to irreversible cell damage or death [9,10,11]. There are many cellular antioxidant defense systems, and in various cardiovascular diseases, other components of the oxidative–antioxidative balance may protect against the development and progression of the disease. From a pathophysiological point of view, ceruloplasmin (CER) and lipofuscin (LPS) may be particularly important in HCM and may be related to the development and progression of HCM. CER is a serum ferroxidase responsible for 95% of copper transport in the blood, and disturbances of copper homeostasis can lead to the hypertrophic cardiac phenotype [12]. Furthermore, CER reduces the bioavailability of nitric oxide (NO) through NO oxidase activity [13], which has also been associated with HCM [14]. CER is also an acute-phase protein, and inflammation can also trigger molecular pathways that contribute to cardiomyocyte hypertrophy and dysfunction [15,16]. In turn, LPS is a pigmented, heterogenous product of intracellular catabolism accumulated mainly in lysosomes, especially in aging cells and pathological conditions [17]. In HCM, the energy metabolism of the myocardium is disturbed, and mitochondrial defects may release excessive amounts of ROS, which may be potentially associated with enhanced lipofuscin accumulation. Furthermore, mitochondrial abnormalities in HCM are related to decreased mitochondrial respiration and the accumulation of damaged mitochondria [18]. 

Taking into account the potential relationship of CER and LPS with the presence of HCM, the aim of this study was to assess the importance of CER and LPS in HCM patients compared to the control group.

## 2. Material and Methods

### 2.1. Study Population

This is single, prospective observational registry enrolling consecutive patients with HCM who were hospitalized in the cardiology department between 2018 and 2021. Patients with recent infection, increased inflammatory markers, liver disease, kidney disease, autoimmune diseases, cancers, smoking cigarettes, or using antioxidant drugs were excluded from the further analysis, leaving 85 patients in the final cohort. Patients with HCM were compared with the control group in terms of basic parameters and oxidative stress markers. The control group consisted of 97 patients who were recruited from the LIPIDOGEN2015 substudy in 2015–2016 and matched for age, the body mass index (BMI), and sex [19]. HCM was diagnosed according to the ESC guidelines for the diagnosis and treatment of hypertrophic cardiomyopathy [20]. The HCM patients were treated optimally using maximum tolerated doses of drugs according to the current recommendations for HCM management [20]. All patients diagnosed with HCM underwent cardiac MRI to exclude other causes of cardiac hypertrophy and to confirm diagnosis of HCM. At the time of enrollment in the study, routine laboratory tests of peripheral blood samples, electrocardiography, echocardiography, and a 6 min walk test were performed in all included patients with HCM. In the control group, patients underwent a history and physical examination to collect data on comorbidities, diet, physical activity, smoking, and family history of heart disease. In all patients, when routine peripheral blood parameters were collected, an additional 5 mL sample was taken to assess oxidative stress parameters.

The study was approved by the Bioethics Committee of the Medical University of Silesia (specific ethics code—KNW/0022/KB1/102/18) and by the Bioethical Committee of the Chamber of Physicians (specific ethics code—KBCz-0018/2015). All included patients gave informed consent to participate in the study.

### 2.2. Laboratory Measurements

Fasting venous samples were obtained after 12 h or more of fasting at the time of enrollment to the study. The complete blood count of patients, as well as hematologic parameters such as hemoglobin concentration and hematocrit, were analyzed using automated blood cell counters (Sysmex XS1000i and XE2100; Sysmex Corporation, Kobe, Japan). Liver and kidney function parameters, cholesterol, and albumin plasma concentrations were determined with a COBAS Integra 800 analyzer (Roche Instrument Center AG, Rotkreuz, Switzerland). The concentration of fibrinogen in plasma was measured using a STA Compact analyzer (Roche Diagnostics GmbH, Mannheim, Germany). A highly sensitive latex-based immunoassay was used to detect plasma C-reactive protein (hs-CRP) using the Cobas Integra 70 analyzer (Roche Diagnostics, Ltd., Mannheim, Germany). The plasma concentration of the N-terminal prohormone of brain natriuretic peptide was measured with a commercially available kit from Roche Diagnostics (Mannheim, Germany) on an Elecsys 2010 analyzer.

The concentration of CER was determined according to the spectrophotometric Richterich method [21]. CER catalyzes the oxidation of colorless p-phenylenediamine to a blue–violet dye. The test sample contained twenty microliters of serum, whereas the control sample contained 20 μL of serum; 200 μL of sodium azide solution was added to stop the reaction. In the next step, 1 mL of p-phenylenediamine dihydrochloride in acetate buffer was added to both samples. After a 15 min incubation, 200 μL of sodium azide was added to the test sample. Finally, after 15 min of incubation, the absorbance of the test and control samples was measured at 560 nm using a PerkinElmer VICTOR-X3 plate reader (Waltham, MA, USA). The intra-assay CV was 3.7%, and the intra-assay precision was 4%. The CER concentration is expressed in mg/dL. 

The activity of superoxide dismutase (SOD) was determined using the method of Oyanagui [22]. A superoxide anion radical (O_2_^−^), produced in the reaction catalyzed by xanthine oxidase, reacts with hydroxylamine to form a nitric ion. Nitric ion combines with naphthalene diamine and sulfaniline acid, producing a colored product. The concentration of this colored product is proportional to the activity of SOD in the samples. The absorbance was read at 560 nm on a Victor X3 Light Plate Reader (PerkinElmer, Shelton, CT, USA). Enzymatic activity was expressed as nitrite units (NU) per milliliter of serum. One NU is defined as 50% inhibition of nitrite ion formation under the method’s condition. The inter- and intra-assay CVs were 2.8% and 5.4%, respectively.

The serum concentration of LPS was determined by Tsuchida et al.’s method [23]. Serum was added to a mixture of ethanol–ether, 3:1 (*v*/*v*), vortexed and centrifuged. The dissolved precipitate was determined by the fluorescence intensity LS45 spectrofluorimeter Perkin Elmer at a wavelength of 345 nm (absorbance) and 430 nm (emission). Values are expressed in relative units (relative lipid extract fluorescence, RF), where an RF value of 100 corresponds to the fluorescence of a solution of 0.1 mg/mL quinidine sulfate in 0.1 N sulfuric acid. LPS concentrations are shown in the RF.

### 2.3. Statistical Analysis

SAS software (version 9.4 SAS Institute, Cary, NC, USA) was used for statistical analyses. Parameters of descriptive statistics for continuous variables with a normal distribution are presented as mean and SD, and variables with a non-normal distribution as median and upper and lower quartiles. Qualitative variables were presented as percentages. Differences between groups were compared using the Student’s t test or Mann–Whitney U test for continuous variables or χ^2^ analysis for noncontinuous variables. The discriminatory power of oxidative stress markers for HCM detection was evaluated by calculating the area under the curve (AUC) from the receiver operating characteristic (ROC) analysis. The optimal cutoff value for the oxidative stress markers was determined using the Youden criterion. The utility of analyzed parameters was evaluated using sensitivity, specificity, and accuracy. A *p* value of <0.05 was considered significant.

## 3. Results

Data of 85 consecutive patients with HCM admitted to our center were analyzed. The control group consisted of 97 patients without HCM. The control subjects and HCM patients were matched for the BMI, age, and sex. The baseline clinical characteristic divided into an HCM group and a control group is presented in Table 1. 

In the patients with HCM, significantly higher CER and LPS levels were observed compared to the control group. There were no differences between the analyzed groups in terms of SOD activity. The comparison of oxidative stress parameters in the HCM and control groups is presented in Table 2. 

**Figure 1:**
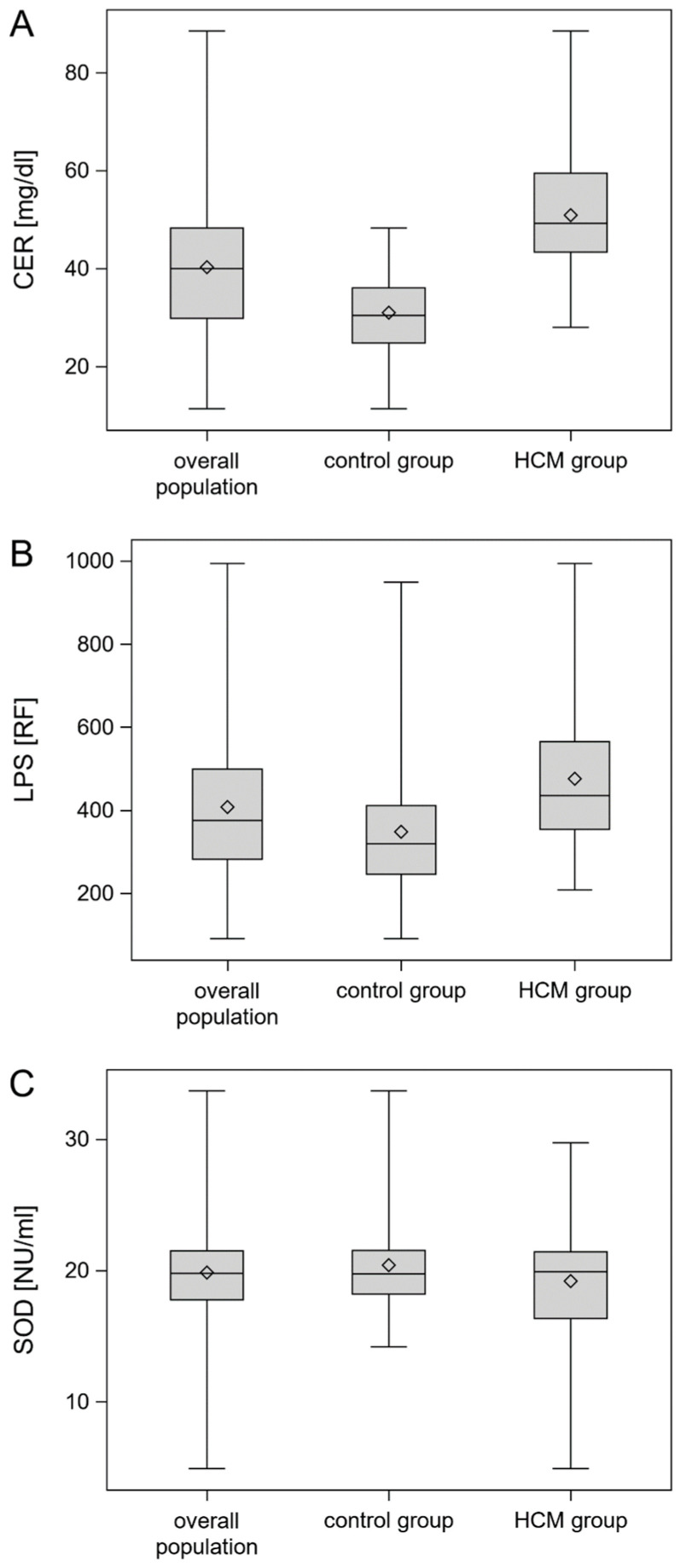
Box with whisker plots of CER (**A**) and LSP (**B**) serum concentrations and SOD activity (**C**). The lower and upper lines of the “box” are the 25th and 75th percentiles of the sample. The distance between the top and bottom of the box is the interquartile range; whiskers represent range of the data. Abbreviations: Abbreviations: CER, ceruloplasmin; LPS, lipofuscin; SOD, superoxide dismutase.

The box with whisker plots of CER and LSP serum concentrations, as well as SOD activity for the HCM group, control group, and overall population, are shown in Figure 1A–C.

Among patients with HCM, 43.5% have LVOT obstruction and 32.9% were diagnosed with systolic anterior motion (SAM). A small percentage of HCM patients underwent alcohol septum ablation or septal myectomy (9.4% and 4.7%, respectively). More than half of the HCM patients (50.6%) were diagnosed with non-sustained ventricular tachycardias. According to the SCD HCM risk calculator, 36.5% of HCM patients were qualified for ICD implantation for primary prevention of sudden cardiac death. Patients received typical treatment for HCM and comorbidities in accordance with the relevant European Society of Cardiology guidelines. The basic demographic, clinical, and laboratory characteristics of patients with HCM are shown in Table 3. 

**Figure 2:**
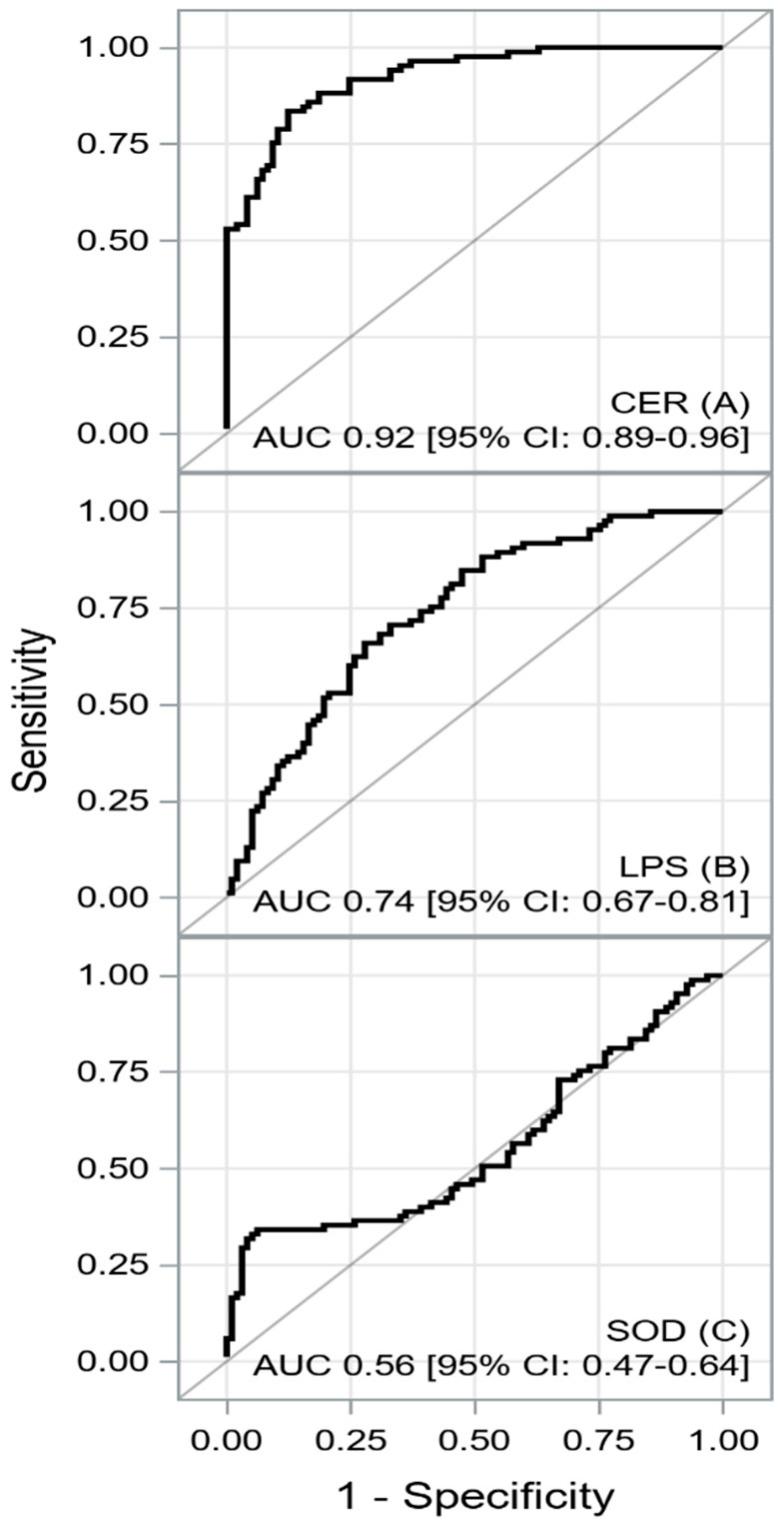
The ROC curves for CER (**A**), LPS (**B**), and SOD (**C**). Abbreviations: AUC, area under the curve; CER, ceruloplasmin; CI, confidence interval; LPS, lipofuscin; SOD, superoxide dismutase.

Among the analyzed oxidative stress parameters, CER had the highest discriminatory power for the detection of HCM combined with good sensitivity and specificity (AUC 0.924, sensitivity 84%, and specificity 88%). LPS had acceptable discriminatory power (AUC 0.740, sensitivity 66%, and specificity 72%). In turn, the utility of SOD for HCM detection was limited (AUC 0.556, sensitivity 34%, and specificity 94%). The ROC curves for CER, LPS, and SOD are shown in Figure 2A–C. 

A summary of the ROC curve analysis for oxidative–antioxidative indicators is presented in Table 4.

## 4. Discussion

Our single-center prospective study demonstrated that higher CER and LPS concentrations in peripheral blood allow for the detection of patients with HCM. CER had excellent predictive power, sensitivity, and specificity for identifying patients with HCM. In turn, LPS had acceptable predictive strength and specificity, as well as limited sensitivity for HCM detection. 

To the best of our knowledge, this is one of the few studies showing that higher CER concentrations are associated with the presence of HCM. The study by Volchegorskiĭ et al. also showed that the serum CER concentration was higher in patients with HCM compared to the healthy volunteers, and physical exercise using the standard bicycle ergometry test caused a significant increase in the CER level in patients with HCM but did not affect the CER level in volunteers [24]. The same authors also demonstrated that the content of CER in the serum reflected tolerance to exercise in the patients with HCM [25]. Potluri et al. also found that HCM was associated with significantly elevated serum copper and CER compared to the matched healthy volunteers [26]. From a pathophysiological point of view, CER may be related to the development and progression of HCM. CER is a multifunctional metalloprotein synthesized mainly in the liver, which transports over 95% of the copper in the body. In turn, copper is an important cofactor of many reactions, and the regulation of systemic metabolism is critical to maintaining proper hemostasis of the body. Abnormal intracellular intercellular transport and defective copper release lead to increased redox reactions and significant release of reactive oxygen species, which may further damage cell molecules [12,27]. Disturbances of copper homeostasis can also lead to hypertrophic cardiac phenotype [12]. Furthermore, CER reduces the bioavailability of nitric oxide (NO) through NO oxidase activity [13]. In turn, in HCM, hearts significantly increased phosphorylated (activated) eNOS, and decreased bioavailability of NO was observed. Further, higher phosphorylation of monomeric eNOS promotes a superoxide anion generation [14], which is one of the more harmful ROS that damages the vascular endothelium and may contribute to increased myocardial stiffness. Downregulation of the NO-cGMP pathway may further lead to hypophosphorylation of sarcomeric proteins, thereby increasing myocardial stiffness and might also contribute to hypertrophy [28]. Furthermore, disturbances of the NO activity promote the production of the peroxynitrite (ONOO^−^), which is capable of oxidizing lipoproteins and nitrating protein-bound and free tyrosine residues [29]. Hassoun et al. also showed that higher 3-nitrotyrosine levels, a marker of peroxynitrite formation, are associated with HCM presence [14]. CER is also an acute-phase copper-binding protein whose concentration increases during inflammation [30]. Inflammation is a well-known pathway in the pathophysiology of cardiovascular diseases. Increasing evidence suggests that inflammation can also trigger molecular pathways that contribute to cardiomyocyte hypertrophy and dysfunction, as well as influence the severity of the phenotype and clinical outcome of HCM. In particular, oxidative stress and inflammation modulate the AKT, ERK1/2, c-Jun, and NO-sGC-cGMP signaling pathways, which are crucial for cardiac function, leading to cardiomyocyte dysfunction, sarcomere damage and, consequently, cardiac hypertrophy [15]. Fang et al. showed that systemic inflammation is associated with parameters of the disease severity in HCM patients, particularly with a degree of hypertrophy and myocardial fibrosis [16].

Another finding of our study is the association of elevated lipofuscin levels in peripheral blood with the presence of HCM. Previous studies have shown elevated LPS levels in end-stage HF due to dilated or ischemic cardiomyopathy, but there are no data about the association of LPS levels with HCM [17,31,32]. LPS is a yellow–brown pigment consisting of a highly oxidized aggregation of lipids, covalently cross-linked proteins, oligosaccharides and metals, which is accumulated mainly in lysosomes, especially in aging cells and pathological conditions [17]. Under conditions of intense oxidative stress, as observed in HCM [5,6,7,8], the accumulation of LPS increases, and ROS produced by damaged mitochondria also contribute to the formation of LPS. Further, LPS, through its ability to incorporate transition metals such as iron and copper, creates an active redox surface that is responsible for increased formation of ROS. Intracellular LPS also interferes with the ubiquitin-proteasome system and the autophagy-lysosomal pathway. These systems are essential for the removal of damaged cells, oxidized proteins and lipids, and impairment of these systems may enhance LPS accumulation and reduce cell viability [33,34]. In an experimental model of rat myocytes was shown that accumulation of lipofuscin is related to the excessive production of H_2_O_2_ by the mitochondria, which penetrates the lumen of lysosomes and interacts with reactive iron through Fenton-type mechanisms, which ultimately leads to the formation of hydroxyl free radicals (OH), inducing lipid peroxidation and ultimately leading to intermolecular cross-linking and lipofuscin formation [35]. It is known that the energy metabolism of the myocardium in HCM is disturbed, and mitochondrial defects may appear in the early phase of the disease and contribute to the progression of HCM [18,36]. Liselotte et al. showed that feline hearts with HCM had a higher mitochondrial ROS release compared to the control group. In turn, increased release of ROS from the mitochondria leads to progressive damage to mitochondrial membrane phospholipids, DNA, and proteins and stimulates cardiac hypertrophy by activation of kinase signaling pathways [5]. Another study also showed that mitochondrial abnormalities in HCM are related to decreased mitochondrial respiration and the accumulation of damaged mitochondria due to increased oxidative stress, decreased antioxidant defense, and lack of upregulation of mitophagic clearance [18]. Considering the fact that lipofuscin accumulation is enhanced by oxidative stress and damaged mitochondria in HCM release excessive amounts of ROS, this may indirectly explain the increased lipofuscin accumulation in HCM patients in our study.

Our study has several limitations. It was a single-center study with a relatively small number of patients with HCM. There was no clarification on whether increased serum CER and LPS concentrations in HCM are the causative factor or a manifestation of the disease. Furthermore, there was a lack of an independent validation cohort that could support our results. The clinical utility of the presented results requires confirmation in larger, multicenter, and prospective studies. Another limitation of the study is the necessity to include multiple selection criteria due to many factors influencing oxidative stress parameters. Moreover, HCM cannot be ruled out with certainty in the control group because screening echocardiography was not included in the study protocol. However, a detailed history and physical examination did not indicate the presence of HCM. Furthermore, the patient population of the LIPIDOGRAM2015 & LIPIDOGEN2015 consisted of consecutive primary care patients [13]; the risk of HCM in this group is a population risk (1:500) and therefore should not significantly influence the results of the study. In addition, we analyzed parameters derived only from peripheral blood. To precisely determine oxidative–antioxidative balance in hypertrophic cardiomyopathy, further studies should be carried out, including blood collection from the heart. Further researches are also needed to understand the molecular mechanism of serum CER and LPS release in patients with HCM

## 5. Conclusions

In summary, our study demonstrates that serum CER and LPS concentrations are significantly elevated in patients with HCM compared to the control group. The simple, inexpensive, and noninvasive indicators of oxidative stress—CER with excellent predictive power and LPS with acceptable discriminatory power—allow for the detection of HCM. There were no significant differences in SOD activity between the analysed group of patients. The present study may have clinical implications in terms of screening for HCM in the general population.

## Figures and Tables

**Table 1:** Characteristics of study population divided into hypertrophic cardiomyopathy group and control group.

	General Population N = 182 ^#^	Control Group N = 97	Hypertrophic Cardiomyopathy Group N = 85	*p* *
Baseline data				
Age, years	53.0 (40–63)	52.0 (40–63)	52.5 (40–63.5)	0.9271
Female, n (%)	107 (58.8)	57 (58.8)	50 (58.8)	0.9934
SBP, mmHg	130 (120–136)	130.0 (120.0–140.0)	125.0 (120.0–130.00)	0.0397 *
DBP, mmHg	80 (70–83)	80.0 (75.0–88.0)	80.0 (70.0–80.0)	0.0002 *
Coronary heart disease, n (%)	33 (18.1)	13 (13.4)	20 (23.5)	0.0769
Hypertension, n (%)	94 (51.6)	52 (53.6)	42 (49.4)	0.5719
Type 2 diabetes, n (%)	3 (1.6)	0 (0)	3 (3.5)	0.1374
Hypercholesterolemia, n (%)	102 (56)	51 (52.6)	34 (65.4)	0.1322
BMI, kg/m^2^	28.3 (4.3)	28.3 (4.1)	28.4 (4.5)	0.8286
HBA1c, %	5.6 (5.2–6.4)	5.5 (5.2–6.0)	5.6 (5.1–6.1)	0.9779

^#^ Data are presented as medians (25th–75th percentile), means (standard deviation), or numbers (percentage) of patients. * *p* < 0.05 (statistically significant). Abbreviations: BMI—body mass index; DBP—diastolic blood pressure; HBA1C—glycated hemoglobin; SBP—systolic blood pressure.

**Table 2:** Markers of oxidative stress in control and hypertrophic cardiomyopathy groups.

Parameter	Control Group *N* = 97 ^#^	Hypertrophic Cardiomyopathy Group *N* = 52	*p* *
SOD	19.7 (18.2–21.5)	19.89 (16.3–21.4)	0.1962
CER	30.4 (24.8–36.0)	49.2 (43.3–59.5)	<0.0001 *
LPS	319.0 (246.0–411.0)	435.05 (353.8–565.0)	<0.0001 *

^#^ Data are presented as medians (25th–75th percentile), * *p* < 0.05 (statistically significant). Abbreviations: CER, ceruloplasmin; LPS, lipofuscin; SOD, superoxide dismutase.

**Table 3:** General characteristics of patients with hypertrophic cardiomyopathy.

Parameters	Hypertrophic Cardiomyopathy Group N = 85 ^#^	Parameters	Hypertrophic Cardiomyopathy Group N = 85
HCM with LVOT obstruction, n (%)	37 (43.5)	TAPSE, mm	22.0 (21.0–26.0)
SAM, n (%)	28 (32.9)	RVSP, mmHg	15.0 (1.0–25.0)
Alcohol septum ablation, n (%)	8 (9.4)	WBC, ×10^9^/L	6.3 (5.3–7.3)
Septal myectomy, n (%)	4 (4.7)	Hemoglobin, mmol/L	8.9 (8.2–9.4)
Coronary heart disease, n (%)	20 (23.5)	Creatinine, µmol/L	87.0 (75.0–106.0)
AF, n (%)	20 (23.5)	Platelets, ×10^9^/L	203.0 (184.0–245.0)
nsVT present, n (%)	43 (50.6)	Total bilirubin, µmol/L	10.30 (7.4–12.8)
HR mean,/min	70.00 (63.00–74.00)	Albumin, g/L	47.0 (45.0–50.0)
ICD, n (%)	31 (36.5)	Uric acid, µmol/L	375.00 (299.0–438.0)
Beta-blokers, n(%)	76 (89.4)	Potassium, µmol/L	4.50 (4.2–4.7)
Cordarone, n (%)	10 (11.8)	Sodium, mmol/L	141.0 (139.0–142.0)
ACEI/ARB, n (%)	39 (45.9)	Fibrinogen, mg/dL	338.0 (291.0–387.0)
MRA, n (%)	19 (22.4)	AST, U/L	23.0 (19.0–31.0)
Verapamil, n (%)	23 (27.1)	ALT, U/L	23.0 (16.0–35.0)
Statin, n (%)	45 (52.9)	ALP, U/L	73.0 (58.0–89.0)
6MWT, n (%)	510.0 (440.0–593.8)	GGTP, U/L	33.00 (21.0–60.0)
IVS max, mm	20.0 (17.0–22.0)	Total cholesterol, mmol/L	4.6 (3.8–5.3)
LVEDD, mm	48.0 (43.0–52.0)	LDL, mmol/L	2.5 (2.0–3.2)
LA, mm	43.0 (37.0–48.0)	hs-CRP, mg/L	1.32 (0.8–2.7)
LVEF, %	55.5 (54.5–63.0)	Ferritin, μg/L	84.0 (37.0–165.0)
RVDD, mm	35.0 (33.0–39.0)	NT-proBNP, pg/mL	333.0 (134.5–863.5)

^#^ Data are presented as medians (25th–75th percentile) or numbers (percentage) of patients. Abbreviations: AF—atrial fibrillation; HCM—hypertrophic cardiomyopathy; HR—heart rhythm; LA—left atrium; LDL—low-density lipoprotein; LVEDD—left ventricular end-diastolic dimension; LVEF—left ventricular ejection fraction; LVOT—left ventricular outflow tract; nsVT—non-sustained ventricular tachycardia; NT-proBNP—N-terminal prohormone of brain natriuretic peptide; RVDD—right ventricular diastolic dimension; RVSP—right ventricular systolic pressure; SAM—systolic anterior motion, TAPSE—tricuspid annular plane systolic excursion; WBC—white blood cells, 6MWT—six minutes walking test.

**Table 4:** A summary of receiver operating characteristic curve curve analysis for biomarkers.

	AUC [±95 CI]	Cutoff	Sensitivity [±95 CI]	Specificity [±95 CI]	Accuracy
SOD	0.556 [0.469–0.643]	<16.74	0.34 [0.24–0.45]	0.94 [0.87–0.98]	0.66 [0.59–0.73]
CER	0.924 [0.887–0.960]	≥40.83	0.84 [0.74–0.91]	0.88 [0.79–0.93]	0.86 [0.80–0.91]
LPS	0.740 [0.669–0.812]	≥389.6	0.66 [0.55–0.76]	0.72 [0.62–0.81]	0.69 [0.62–0.76]

Abbreviations: AUC, area under the curve; CER, ceruloplasmin; CI, confidence interval; LPS, lipofuscin; SOD, superoxide dismutase.

## Data Availability

The data presented in this study are available on request from the corresponding author. The data are not publicly available due to privacy restrictions related to the rules in our institution.
